# Reflective Functioning, Emotion Regulation and Physiological Reactivity in Children with and without Behavioral Disorders: a Multimethod Approach

**DOI:** 10.1192/j.eurpsy.2025.1179

**Published:** 2025-08-26

**Authors:** M. Tironi, A. Schiavoni, F. Rospo, F. Bizzi, S. Charpentier Mora

**Affiliations:** 1 University of Genoa, Genoa, Italy

## Abstract

**Introduction:**

Mentalizing (operationalized as reflective functioning; RF), emotion regulation (ER), and reactivity (operationalized as physiological reactivity; PR) are highly relevant protective factors during development. However, limited research has examined these constructs during middle-childhood using a multimethod approach.

**Objectives:**

The first aim compared differences in these constructs between children with behavioral disorders and a non-clinical group, measuring PR during a dyadic stress task involving conflict with the mother. The second aim explored the relationship between RF and both ER and PR considering the moderating role of externalizing symptomatology.

**Methods:**

The study involved 50 children with behavioral disorders (*Mage* = 11.3, *SD* = 1.76; 58% male) and 89 non-clinical children (*Mage* = 10.6, *SD* = 1.64; 48% male). The *Child Reflective Functioning Scale* applied to the Child Attachment Interview was used to assess RF, the *How I Feel* to assess self-reported ER over the past three months, and the *Positive and Negative Affect Scale for Children* to assess self-reported ER before and after the dyadic stress task. Shimmer 3 GSR+ device has been applied to measure physiological indexes of heart rate variability (HRV) and galvanic skin response (GSR) to assess PR during the dyadic stress task. Mothers completed the *Child Behavior Checklist* to assess child’s externalizing symptomatology.

**Results:**

Children with behavioral disorders showed lower levels of both global and others-oriented RF and reported higher levels of negative emotions in the previous three months, compared to their non-clinical peers. Both global and others-oriented RF were negatively correlated with reported negative emotions. In contrast, the non-clinical group exhibited higher levels of positive emotions in the previous three months and greater PR during the dyadic stress task. Additionally, externalizing symptomatology moderated the association between others-oriented RF with 1) physiological reactivity (i.e., GSR), only at lower levels of externalizing symptoms; 2) emotion regulation (i.e., emotional control subscale of the *How I Feel*), only at higher levels of externalizing symptomatology.

**Conclusions:**

These findings reinforce prior research suggesting that children with behavioral disorders exhibit lower levels of RF, especially towards others, and negative emotions which may be risk factors for the development of behavioral disorders. Using a multimethod approach allowed to both evaluate ER and PR, highlighting the differences between the subjective perception of emotions and their physiological response. Lastly, the lower PR during the dyadic stress task in the clinical group could be a consequence of greater habituation to conflict, a hypothesis that could be explored in future research.

**Disclosure of Interest:**

None Declared

